# State Board of Health of West Virginia

**Published:** 1882-12

**Authors:** 


### STATE BOARD OF HEALTH OF WFST VIRGINIA.

The following circular, received from the Secretary of the Board, will give our readers a fair knowledge of the scope and practical working of the recently enacted laws of West Virginia in relation to the Public Health and Medical Practice. ED.

OFFICE OF THE STATE BOARD OF HEALTH.
WHEELING, September, 1882.

TO LOCAL BOARDS OF HEALTH, PHYSICIANS AND OTHERS INTER-TERESTED IN SANITARY SCIENCE.

*Gentlemen:* The law establishing a State Board of Health and regulating the Practice of Medicine and Surgery, passed March 8. 1881, and amended and re-enacted March 15, 1882, is now in full force and effect in every county of the State, and the earnest co-operation of all persons asked who desire that human suffering shall be alleviated, the death-rate diminished, and the sanitary conditions of our State improved. Without such hearty co-operation, especially on the part of local boards of health and the members of the medical profession generally, the law, however wise in its provisions, must fail utterly in conferring upon the whole people of West Virginia the humane and priceless benefits contemplated by its passage.

All who engage in the study of local conditions affecting the public health—the influence of soil, climate, water and food supplies, drainage and sewerage; the influence of race, population and social conditions; or who watch the invasion of contagious and infectious diseases to discover the agencies or causes which produce and maintain them, and devise the proper methods for their control; who assist in collecting reliable vital statistics and in any wise employ their energies to systematize and perfect the grand work intrusted to those in charge of the public health—are public benefactors.

It is an encouraging sign of the times that so much attention is now being paid by lawgivers to the prevention of disease. The art of the physician can do much in curing disease in detail, but the power of a State legislature, if well directed, may do infinitely more in adopting and enforcing measures to check, if not wholly prevent, the development of a large class of very serious maladies.

During the last century, statistical researches have developed two extremely important facts, namely, that the mean duration of human life is generally less than half that of the three score and ten years commonly allotted as the term of man's existence, but that, on the other hand, communities have it, to a certain degree, in their power to diminish the causes by which the lives of their members are thus abridged ; and it has been made evident that, in consequence of the judicious employment of that power, the average duration of human life has been for many years slowly but progressively on the increase.

Stimulated by the activeness of sanitarians the public mind is becoming more and more enlightened as to the connection of *filth* and *disease;* and it is the duty of local boards of health to busy themselves to the end that such knowledge shall pervade and influence every household in the State.

### THE APPOINTMENT OF LOCAL BOARDS AND THEIR DUTIES.

By reference to sections 6 and 7 of the amended act of 1882, it will be seen that local or county boards have a wide and responsible field of duties.

The manner of appointing local boards under the law of 1882 precisely reverses the method prescribed in the act of 1881. Now it is simply the duty of the county court to nominate for each county "three intelligent and discrete persons residing therein, one of whom, at least, shall be a person legally qualified to practice medicine," and the State Board may or may not confirm the nominations so made by the court. In the event of non concurrence by the State Board, it is the duty of the court to name other persons for the office, and so continue to nominate until the State Board shall accept and appoint. In covering the State with local boards of health, there has been no serious conflict of authority in the matter of appointments except in a single instance; and this speaks well of the favor with which the passage of the law was received, and the pleasure of county courts in putting into effect the most useful arm of its power.

Persons appointed on local or county boards of health hold their office for the term of two years, unless sooner removed by the State Board. As soon as possible after their appointment, the board shall meet for organization—the member first named on the list of appointments making the call—and elect from their number a presiding officer as prescribed in section 6 of the act. The county board thus organized "shall make and establish for the county, or for any place or district therein, such sanitary rules and regulations as may be deemed necessary and proper by the said board to prevent the outbreak and spread of infectious or contagious diseases."

"The local board of any county may declare quarantine therein, or in any particular district or place therein, against the introduction of any contagious or infectious disease prevailing in any other State, county or place, and of any and all persons and things likely to spread such contagion or infection." But "as soon as such quarantine is established, the local board shall, in writing, inform the members of the State Board residing in that congressional district, whose duty it shall be to ascertain the necessity of such local quarantine and take action accordingly."

In the matter of expenditures of money necessary for local and county sanitation, and of all bills incurred by local boards of health, their agents or appointees, while in the performance of official duty, the pleasure of the county court must be consulted. "In cases of emergency, or of actual necessity, and when the court or corporate authorities are from any cause unable to meet or to provide for the emergency or necessity of the case, all actual expenditures shall be certified by the local board to the county court, and the whole or as much thereof as the said court may deem right and proper, shall be paid out of the county treasury." It is plain, therefore, that the most intimate and reciprocal relations should exist and be cultivated between county courts and local boards of health in order that there shall be no lack of energy in the performance of sanitary work, on the one hand, nor liberality in the payment of just bills for service rendered, on the other. A careful study of sections 6 and 7 of the amended act will show direction for every duty to be performed by local boards.

THE DUTY OF PHYSICIANS.

The law makes it the plain duty of every practicing physician to report promptly to the presiding officer of the local board of his county all cases of cholera, small-pox, scarlet fever, diphtheria, enteric or typhoid fever, whooping cough, measles or other endemic, epidemic, infectious or contagious diseases of dangerous character, under treatment by him. To encourage and facilitate a full and prompt report by physicians, cards in blank will be distributed to them through local boards. In this way the duty of making report will be made easy, and the most thorough sanitary police of the State secured. The result of this required labor of physicians will soon be seen in reports compiled therefrom by local boards whose duty it shall be report to to the State Board of Health "once, at least in every three months, the character of all such infectious, contagious, endemic or epidemic diseases; the number of persons reported as affected with either of said diseases, naming the same; the action taken by the local board to arrest the progress of every such disease, and the visible effects (if any) of such action."

"Where any city, town or village has a board of health of its own, the jurisdiction of the local or county board shall not extend thereto, but such city, town or village board shall be auxiliary to and act in harmony with the State Board of Health."

### THE PRACTICE OF MEDICINE.

It is remarkable and worth telling that the State Board of Health has had trouble in but *three* cases among the thousand and sixty applicants for medical certificate. Indeed, nothing could work more smoothly than that part of the machinery of the law regulating the practice of medicine and surgery, for it has the hearty support of every respectable physician in the State, and all are proud of our rising professional standard.

The new manner of dealing with itinerant physicians has not only put several hundred dollars into the State treasury, but prevented the incursions of a class of ignorant pretenders who, previous to the passage of the law, regularly occupied West Virginia to humbug credulous people.

It is the duty of every legally qualified physician to keep close watch for all violations of the law, and when offenses are discovered, to report them promptly to the prosecuting attorney or to a justice of the peace.

*Section* 14 makes it the duty of the sheriff to collect the "special tax of $50 for each month and fraction of a month" from all *itinerant physicians*, of whatever name or character, "who shall travel from place to place, and by writing, printing or otherwise, publicly profess to cure or treat diseases, injuries or deformities." Any person is regarded as practicing medicine within the meaning of the law "who shall publicly profess to be a physician, and to prescribe for the sick, or who shall append to his name the letters 'M. D.'"

The act also applies "to druggists and pharmacists who prescribe for the sick," and should be enforced against such offenders to the letter. Frequent complaints have reached the State Board of violations of the law by druggists and pharmacists, and there is good reason to believe that such offenses, though not as common as before the passage of the law, are yet of frequent occurrence in many of our large towns. By all proper means, such offenders should be discovered, and the information given to the prosecuting attorney or a justice of the peace.

Finally: The law requires that all persons engaged in the practice of medicine and surgery in West Virginia shall have a certificate from the State Board of Health. There are three classes of certificates: 1st, the diploma class; 2nd, the ten years' experience class; 3d, the examination class. "Itinerant physicians" must possess the qualification of one or the other of these classes before offering to engage in general practice or to prosecute a specialty; and unless they first so provide themselves with a medical certificate from the State Board they become offenders in a *double sense* and liable to the severest penalties of the law.

Should the registration of any person engaged in the practice of medicine be questioned, or doubtful, specific information may be had by addressing the office of the secretary of the State Board of Health, giving the name in full, as well as the residence of the supposed offender.

### THE STATE BOARD WILL CO-OPERATE WITH THE LOCAL BOARDS OF HEALTH.

☞ By immediately answering all communications from local boards.

By sending brief suggestions to meet any emergency, or when the public health is endangered from any cause.

By telegraphing and writing to local authorities to secure the best means of protection; or by visiting in person the county, district, or place, for the purpose of instituting and enforcing rules for the suppression of infectious or contagious diseases prevailing therein.

By supplying all needful forms for reports by physicians, and giving such information to local authorities as shall be necessary for the protection of the public health.

☞ THE STATE BOARD advises that every local or county board be prepared against small-pox, by urging all school boards to prohibit the attendance of un-vaccinated children; by an agreement with all physicians to vaccinate every unprotected person in each household where they attend; by instructions to all health officers and agents or officers of local boards to ascertain who in each community are unvaccinated and exposed to small-pox; by notifying the secretary of the State Board of Health without delay when small-pox appears; by writing or telegraphing the situation of the case or cases; and by requiring prompt compliance with all orders issued either by the local or State Board for the control of disease.

The State Board urges upon local or county boards the importance and necessity of a prompt and careful distribution of the blank cards sent from this office for the use of physicians in making reports of all infectious, contagious, endemic or epidemic diseases under treatment by them, in obedience to section 6 of the amended act. Complete lists of the registered physicians of any county will be furnished on application to the Secretary of the State Board; and local boards should regard it one of their special duties to require of all physicians complete returns according to law.

The accompanying postal card, addressed to the Secretary of the State Board, is for the acknowledgement of the receipt of this circular and other documents sent with it. On it should also be returned the name in full and post-office address of each member of the local or county board of health, not omitting to distinguish the presiding officer, and the secretary, if there be such officer. The postal card should be returned promptly, in order that the record of local boards shall be correct in every particular.

Trusting that this circular may serve to increase the confidence of the medical profession in the State Board, and lead to reforms that shall advance the cause of the public health in West Virginia, I am, Your obedient servant,

JAMES E. REEVES, M. D.,
*Secretary State Board of Health.*

MEMBERS OF THE STATE BOARD OF HEALTH.

Geo. B. Moffett, M.D., President, Parkersburg; James E. Reeves, M.D., Secretary, Wheeling; C. T. Richardson, M.D.,

Charlestown, Jefferson County; Geo. H. Carpenter, M.D., Moorefield, Hardy County; Hon. A. R. Barbee, M.D., Point Pleasant; Gabriel McDonald, M.D., Union, Monroe County; Lawrence Carr, M.D., Charleston, Kanawha County; Wm. M. Late, M.D., Bridgeport, Harrison County.

